# Effects of Fiber Surface Grafting with Nano-Clay on the Hydrothermal Ageing Behaviors of Flax Fiber/Epoxy Composite Plates

**DOI:** 10.3390/polym11081278

**Published:** 2019-07-31

**Authors:** Anni Wang, Guijun Xian, Hui Li

**Affiliations:** 1 Key Lab of Structures Dynamic Behavior and Control of the Ministry of Education, Harbin Institute of Technology, Harbin 150090, China; 2 Key Lab of Smart Prevention and Mitigation of Civil Engineering Disasters of the Ministry of Industry and Information Technology, Harbin Institute of Technology, Harbin 150090, China; 3 School of Civil Engineering, Harbin Institute of Technology, Harbin 150090, China

**Keywords:** flax fiber, nano-clay, water uptake, hygrothermal properties

## Abstract

Flax fiber has high sensitivity to moisture, and moisture uptake leads to the decrease of mechanical properties and distortion in shape. This paper attempts to graft flax fabric with nano-clay, with assistance from a silane-coupling agent, in order to improve hygrothermal resistance. The nano-clay grafted flax fabric reinforced epoxy (FFRP) composite produced through vacuum assisted resin infusion (VARI) process were subjected to 80% RH chamber for 12 weeks at 20, 40 and 70 °C, respectively. Moisture uptake, dimensional stability, and tensile properties was studied as a function of humidity exposure. Through SEM and FTIR, the effects of hygrothermal exposure was elucidated. In comparison to control FFRP plates, nano-clay grafting decreases saturation moisture uptake and the coefficient of diffusion of FFRP by 38.4% and 13.2%, respectively. After exposure for six weeks, the retention rate of the tensile modulus of the nano-clay grafted flax fiber based FFRP increased by 33.8% compared with that of the control ones. Nano-clay grafting also reduces the linear moisture expansion coefficient of FFRPs by 8.4% in a radial direction and 10.9% in a weft direction.

## 1. Introduction

Flax fiber is a natural fiber that is biodegradable, renewable and environment-friendly compared to traditional carbon fiber and glass fiber. Flax fiber possesses relatively higher tensile strength compare to other natural fibers, which are considered as a high performance natural fiber. Although its water resistance properties are not very good compared to thermoplastic polymer, epoxy resin has been widely used as a resin matrix for polymeric composites. Epoxy resin has good wettability with flax fiber, which provides good interface properties of flax fiber reinforced epoxy polymer (FFRP) composites. FFRPs are widely used in decorative materials, automobiles and other fields due to their high specific modulus. However, due to the high hydrophilicity of flax fibers, their poor durability limits the development of FFRPs.

The special chemical composition and structure of flax fiber lead to high hydrophilicity. Flax fiber is composed of plant cells, whose main structure are cell walls [1,2]. Like most plant cells, flax fiber cell walls consist mainly of cellulose, hemicellulose and pectin [3,4,5]. Cellulose, hemicellulose and lignin are made up of macromolecular chains of glucose and contain a large number of hydroxyl groups, which can adsorb water molecules [6]. Cellulose, called micro fibrils, is wrapped by hemicelluloses and lignin, and glued together or linked by hydrogen bonds [7]. Crystalline cellulose cannot store water, but water molecules can store them inside the amorphous hemicellulose and lignin [8,9]. In addition, flax fiber cells contain cell cavities that can store water. Thus, compared to traditional fiber such as carbon fiber, flax fiber shows high water absorbability.

Owing to the water absorption of fibers and the storage of water molecules at the interface between fiber and polymer, FFRPs also exhibit high water absorption [10,11]. Researchers have done a lot of research on the water absorption process of nature fiber reinforced polymer composites (NFRPs). The water absorption process of NFRPs is consistent to Fick law at lower temperature [12,13,14]. Both saturated water absorption and rate of water absorption of NFRP composites samples increase as the fiber volume fraction increase [14,15]. At the same time, the diffusion rate of water molecules in NFRP is related to temperature. The higher the temperature, the faster the diffusion rate. The researchers also studied the deterioration of mechanical properties of NFRP in a hygrothermal environment. On the other hand, the absorbed moisture results in more detrimental effects on the mechanical properties of NFRPs since the water not only interacts with fiber and polymer matrices, physically, i.e., plasticization, and/or chemically, i.e., hydrolysis, as in the unfilled system, but it also attacks the fiber–matrix interface [10]. Thus, the decrease of mechanical properties of NFRP caused by water molecules entering composite materials. Hongguang Wang et al. put the ramie fiber reinforced composites at 20 ℃ and 40 °C under 100% RH, and found that only after 1 day, both of the flexural strength and modulus were reduced dramatically and the deterioration rate of strength and modulus slowed down with the extension of immersion time [16].

Surface treatment of fiber is a good way to improve the properties of FRPs by improving the interface properties [17,18,19]. In order to promote the application of natural fiber, its hygrothermal resistance properties need to be improved by fiber treatment. At present, the main methods to improve the hygrothermal properties are as follows: removing the active hydroxyl groups on the surface of the fibers by chemical reaction, reducing the adsorption sites of water molecules; coating hydrophobic coatings on the surface of the fibers, hindering the diffusion of water molecules in the fibers. H. Alamri et al. used n-SiC fill cellulose fiber reinforced epoxy eco-nanocomposites and found that saturated water absorption of the composites decreased with the increase of n-SiC content [20]. Anna Dilfi K.F. et al. studied the durability of jute fiber reinforced epoxy composites treated by alkali and silane coupling agents and found that the deterioration of mechanical properties of jute fiber reinforced composites after chemical treatment are less than that of untreated ones [21]. Gao Ma et al. found that both alkali and silane treatments of jute fiber reduced water absorption and enhanced the tensile strength of the resulting jute fabric/epoxy composites [22].

The hydrophobicity and lamellar structure of nano-clay makes it possible to improve the durability of flax fibers after grafting onto the surface of flax fibers. Nano-clay is a kind of special nano-material with a large specific surface area, which is composed of two tetrahedral silicon atoms and eight sides of aluminum or magnesium hydroxide [23]. Nano-clay exhibits a hydrophobic lamellar structure, so polymer-clay nanocomposites have received much attention due to significant increase in mechanical properties, and a moisture barrier [24]. Polymer nanocomposites contain relatively small amounts (typically less than 5 wt. %) of nanometer-sized filler particles, which, if properly dispersed, have been found to cause significant reductions in both gas and water vapor permeability [25]. Neetu Malik et al. mixed biodegradable polymer polycaptalactone (PCL) and organic modified montmorillonite clay (OMMT) and found that with an increase in weight percentage of OMMT within the bio polymer films, the moisture absorption value of bio-nanocomposite films reduced rapidly from 34.4% to 22.3% [26].

In this paper, the method of improving the durability of flax fiber reinforced composites by nano-modification of flax fiber is studied. The effects of nano-clay grafting on the water absorption process as well as deterioration of mechanical properties of the related FRPs in hygrothermal environment were investigated. The mechanism of surface grafted nano-clay on durability of FFRPs was studied.

## 2. Experimental

### 2.1. Materials

The organic nano-clay, belonging to a high purity montmorillonite organic ammonium derivative, was purchased from Lingshou Huarun Mineral Factory (Shijiazhuang City, China). The nano-clay used in the present study is an organically treated montmorillonite (OMMT) with ammonium. Bi-directional flax fiber fabrics was purchased from Harbin Flax Textile Co., Ltd., (Harbin, Heilongjiang Province, China). The density of fabric is 1.5 g/cm^3^ and nominal thickness is 0.16 mm. The silane coupling agent used in the current work is 3-Triethoxysilylpropylamine (APTES, KH550), purchased from Chengong Silicon Company (Nanjing, China) with purity of 98%. The epoxy resin used is room temperature impregnating adhesive (TS), purchased from Shandong Dagong Composite Material Co., Ltd (Linyi, China).

### 2.2. Surface Grafting of Flax Fabric

**Figure 1:**
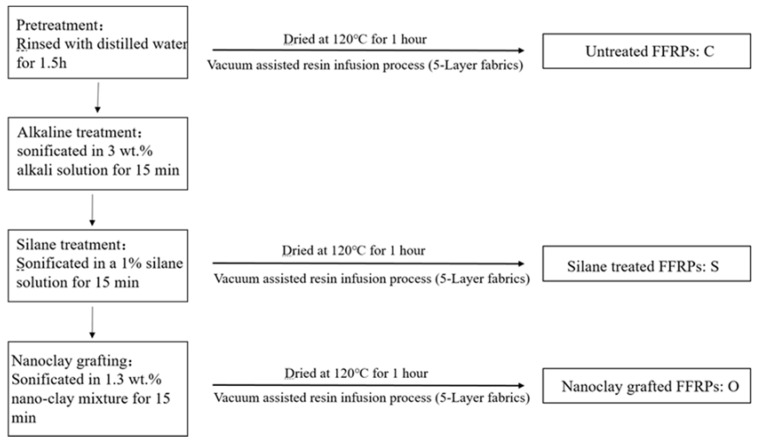
Methods of nano-clay grafted onto flax fiber and preparation of flax fiber reinforced polymer composites (FFRPs).

According to the author’s previous work [27], the preparation process of nano-clay grafted flax fiber/epoxy resin composite is shown in Figure 1. The details can be found in Ref. [28]. Nano-clay were dispersed into a solvent (ethanol: distilled water = 4:1 by weight) with an ultrasonic bath. The organic nano-clay content in the dispersion medium is 1.3 wt. %. After 1 h of ultrasonic treatment at room temperature, flax fabric and 1wt % KH550 (to the dispersion medium) was added to the solution and sonificated for 15 min at room temperature. Finally, the fiber was washed in distilled water for 5 min. The composites were prepared by vacuum-assisted resin infusion process.

**Figure 2:**
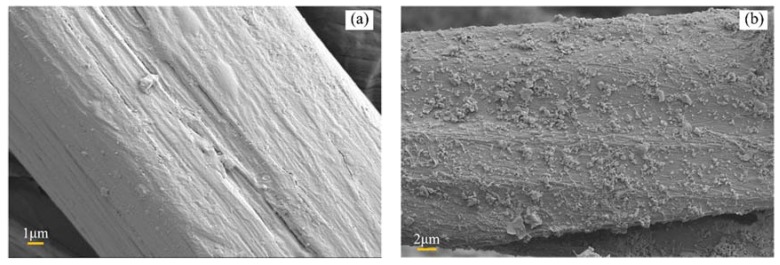
SEM photos of flax fiber: (**a**) untreated fiber (**b**) nano-clay grafted fiber.

Figure 2 shows the SEM pictures of untreated fiber and nano-clay grafted flax fiber. When compared to the untreated one, lamellar nano-clay can be seen on the surface of the flax fiber. Due to its hydrophobicity, the presence of nano-clay enhances the barrier properties of the materials by creating tortuous pathways for water molecules to diffuse into flax fiber, which leads to a reduction in absorbed water and the coefficient of diffusion. Lamellar and hydrophobic nano-clays inhibit the diffusion of water molecules more obviously than other nano materials.

### 2.3. Hydrothermal Environment Conditions

**Figure 3:**
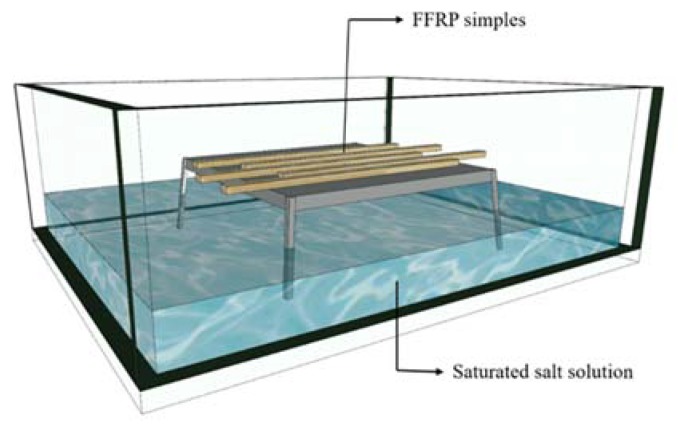
Schematic diagram showing preparation of a hydrothermal environment with a saturated salt solution.

In the experiment, three different humid and hot environments were prepared using a saturated salt solution (Table 1). At 20 ℃ and 40 °C, the saturated potassium bromide solution can have ambient humidity of 80 RH%. At 70 °C, the humidity is 80% with saturated potassium chloride solution. As shown in Figure 3, the humidity chamber is prepared with a saturated salt solution in glass boxes at different temperatures. The humidity and temperature in the sealed glass box are monitored. When the temperature and humidity are stable, FFRP samples are placed in the sealed box. The condensed water droplets are prevented from falling on to the surface of the sample during the experiment. Before placing into the hygrothermal environment, the FFRP samples were dried in an oven at 70 °C for 48 h.

### 2.4. Characterization

#### 2.4.1. Moisture Uptake

According to ASTM D5229, the sample for moisture uptake is 76 mm × 25 mm, more than 5 g. Each group contains eight samples. Moisture uptake was detected by periodically recording the mass of the sample. Samples taken out of the humidity chamber were weighed using an electronic balance with accuracy of 0.01 mg. The presented data are an average for eight coupons. The immersion periods were set as 4 h, 8 h, 12 h, 1 day, 2 days, 4 days, 1 week, 2 weeks, 3 weeks, 4 weeks, 5 weeks, 8 weeks and 12 weeks.

#### 2.4.2. Fourier Transform Infrared Test

Flax fiber fabrics were cut into powder—about 2 mg—to make fiber specimens. Fourier transform infrared (FTIR) spectra of the fiber specimens (control and treated flax fiber yarn) were recorded on a spectrometer (Spectrum 100, Perkin Elmer Instruments, Boston, MA, USA) at a range of 400–4000 cm^−1^.

#### 2.4.3. FBG Monitoring

Fiber Bragg Grating (FBG) Demodulator is manufactured by Shanghai Qipeng Engineering Materials Technology Co. (Shanghai, China) The FBG sensor for temperature monitoring was encapsulated in a steel capillary. With protection of the steel capillary, the FBG sensor was in a strain-free condition and affected only by the temperatures. To measure the internal stress during the aging, the FBG sensors were embedded in the interlayers of the FRP wet layups. The FBG sensors were carefully located in the fiber direction or perpendicular to the fibers and placed in the middle of the plate along the depth of the layer and near the central part in the planar direction. The fabricated resin samples were cured with the same curing procedure as the FRP samples. In this work, FFRPs were embedded with two FBG sensors—one along the fiber direction and one in the perpendicular direction. The fellow equations describe the strain measurement with the FBG sensors:

(1) $$\Delta \lambda =\Delta \lambda_{1}+\Delta \lambda_{2}$$

(2) $$\Delta \lambda_{1}=\alpha_{\varepsilon }\Delta \varepsilon$$

(3) $$\Delta \lambda_{2}=\alpha_{T}\Delta T$$

where Δλ is FBG wavelength change; Δλ_1_ is strain-induced FBG wavelength change; Δλ_2_ is temperature-induced FBG wavelength change; $\alpha_{\varepsilon }$ is strain sensitivity coefficient, which is 1.2 pm/με for the FBG used in this experiment; $\alpha_{T}$ is temperature sensitivity coefficient, which is 10 pm/°C for FBG used in this experiment; Δε is strain value; ΔT is temperature change.

The wavelength change obtained by the FBG can be converted into strain:

(4) $$\Delta \varepsilon =\frac{\left(\Delta \lambda -\Delta \lambda_{2}\right)}{\alpha_{\varepsilon }}$$

#### 2.4.4. Mechanical Property Test

Tensile properties of the FFRP plate samples were tested according to ASTM D3039. The dimensions of the specimens were 250 mm × 15 mm. The crosshead speed is set as 2 mm/min. The samples were removed from the hygrothermal environment chamber at regular time intervals, i.e., 2, 4, and 6 weeks. Five samples were repeated for one condition.

#### 2.4.5. Scanning Electron Microscope (SEM) Test

For the SEM test, all the specimens were sputter coated with gold for 15 min (Gatan Model 682 Precision etching coating system) before SEM analysis to improve their electrical conductivity. The control and treated FFRP samples were observed through a scanning electron microscope with accelerated voltages of 20–30 V.

## 3. Results and Discussion

### 3.1. Moisture Absorption of FFRPs 

The water absorption percentage of FFRPs in hydrothermal environments can be calculated by the following equation:

(5) $$\Delta M\left(t\right)=\frac{m_{t}-m_{0}}{m_{0}}\times 100$$

where Δ*M* is moisture uptake, *m*_0_ and *m_t_* are the mass of specimen before and after exposure time of *t*.

**Figure 4:**
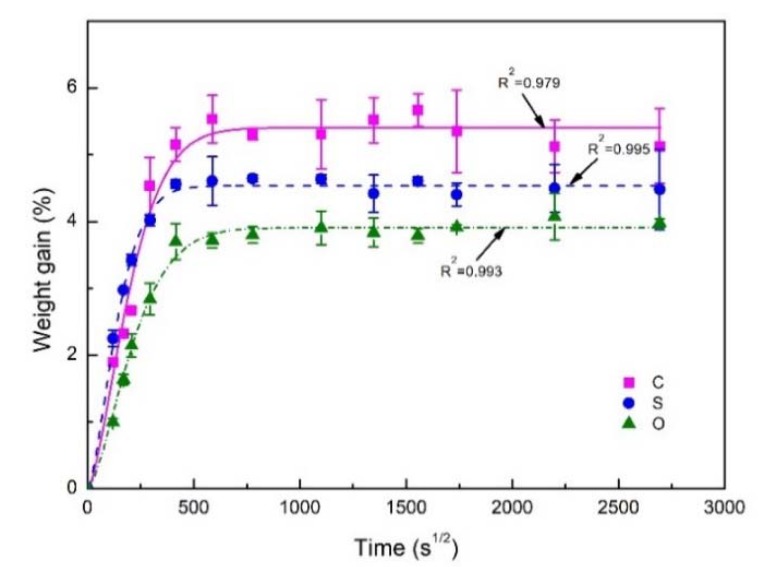
Water absorption process of FFRPs at 70 °C under 80% RH. (C: untreated FFRPs; S: silane coupling agent treated FFRPs; O: nano-clay grafted FFRPs).

Figure 4 shows the percentage of weight gain of untreated (C), silane coupling agent treated (S) and 1.3 wt. % nano-clay grafted flax fiber reinforced epoxy composites (O) at 70 °C under 80% RH from 0 to 3 months. Fick’s diffusion model describes the dynamic equilibrium of the water absorption process when the material reaches a certain degree of water content. In the initial stage, the material’s water absorption is proportional to the square root of exposure time. With the passage of time, the moisture absorption rate decreases dramatically, and finally reaches a dynamic balance. As shown in Figure 4, the moisture uptake process for all samples is linear in the beginning, then levels off, indicating a Fick diffusion process. R^2^ represents the deviation between the experimental data and fitting results with the Fick’s model.

According to Fick’s law, the diffusion of water in materials is controlled by the concentration gradient of diffused substances. To obtain the water uptake and diffusion parameters, the curve fitting method was adopted with two-stage water uptake models:

(6) $$M_{t}=M_{m}\left\{1-exp\left[-7.3{\left(\frac{Dt}{h^{2}}\right)}^{0.75}\right]\right\}$$

where $M_{m}$ is the maximum weight gain, $M_{t}$ is the weight gain at time *t* and h is the half of thickness of the composite.

Using Equation (2), the water diffusion parameters of the FFRPs are obtained as shown in Table 2. The nano-clay grafted flax leads to a remarkable decrease in saturated moisture uptake by 38.4% and 15.4% compared with the control and silane-treated ones. Similarly, nano-clay grafting also results in the reduction of the diffusion coefficient by 13.2% and 56.6% than that of the control and silane-treated ones. It is worth noting that silane-treated FFRPs show higher coefficient of diffusion and lower water uptake compared with the untreated ones. The lower water uptake of silane treated FFRPs is attributed to the reduced hydroxyl groups of flax fibers, which were reacted with silane. Note that the silane reaction is not complete, and some silane coupling agents did not form a network. Those relatively low molecules existing between the fiber and resin matrix accelerate water absorption. As a result, the FFRPs with silane-treated FFRPs show a higher coefficient of diffusion.

**Figure 5:**
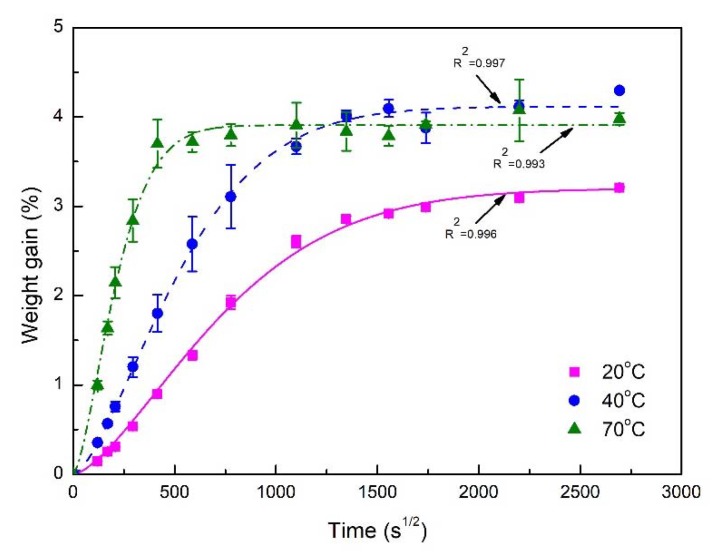
Water absorption process of nano-clay grafted FFRPs at 20 ℃, 40 ℃ and 70 °C under 80% RH.

The water absorption process of nano-clay grafted FFRPs is also affected by temperature. Figure 5 shows the water absorption process of nano-clay grafted FFRPs at 20, 40 and 70 °C under 80% RH, which all followed a Fickian diffusion process. After aging for three months, the samples at 40 and 70 °C were saturated with water and had the same saturated water absorption, while the samples at 20 °C under 80% RH reach saturated water absorption and had lower saturated water absorption. As shown in Table 3, with the increase of temperature, the diffusion rate of water molecules increases. As the temperature increases, the higher the activation energy of water molecules is, and the faster is water saturation of FFRPs. Ana Espert et al. got the same results by immersion of wood fibers/polypropylene composites in water at three different temperatures [14].

### 3.2. FTIR Observation

**Figure 6:**
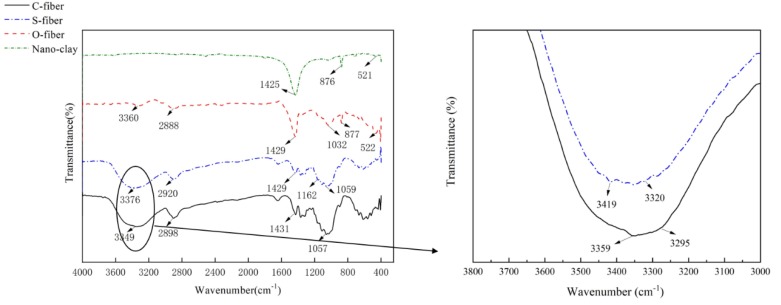
FTIR test results of flax fiber (C-fiber: untreated fiber; S-fiber: silane treated fiber; O-fiber: nano-clay grafted fiber) [24].

As shown in Figure 6, FTIR test results display the fundamental OH stretching vibration of untreated fiber and silane treated fiber [20]. In the FTIR results of untreated flax fibers, the peak at 3295 (cm^−1^) indicated hydroxyl groups adsorbed by hydrogen bonds and the peak at 3359 (cm^−1^) indicated free hydroxyl or amino groups. Because the untreated flax fibers contain no amino group, this peak completely represents the vibration of hydroxyl group [28,29]. FTIR results of silane coupling agent treated fiber showed that the peak at 3320 (cm^−1^) represented hydroxyl groups adsorbed by hydrogen bond, and the peak at 3419 (cm^−1^) represents free hydroxyl or amino groups [28,29]. Because silane coupling agent treated flax fibers contain a large number of amino groups, and the absorption peaks become wider, indicating that there are overlapping groups, free hydroxyl and amino groups are represented here. Because the hydroxyl absorption peaks of the two fibers are similar, it can be considered that the hydroxyl content of silane treated flax fibers is lower than that of untreated flax fibers. The saturated water absorption of silane-treated FFRP decreases due to the decrease of hydroxyl content and the decrease of water adsorption sites. This phenomenon also can be explained by the chemical reaction between flax fibers and nano-clay. Based on previous research by the author, the interface properties of flax fiber and resin was improved using this chemical treatment. Reduction of water absorption could be attributed to the complete adhesion and wettability between the flax fibers and the polymer matrix, which may have less gaps and flaws at the interface [30]. The increase of interfacial bonding reduces the amount of water molecules stored at the interface of FFRP [10]. This chemical reaction was presented in a previous research work by the author [27].

**Figure 7:**
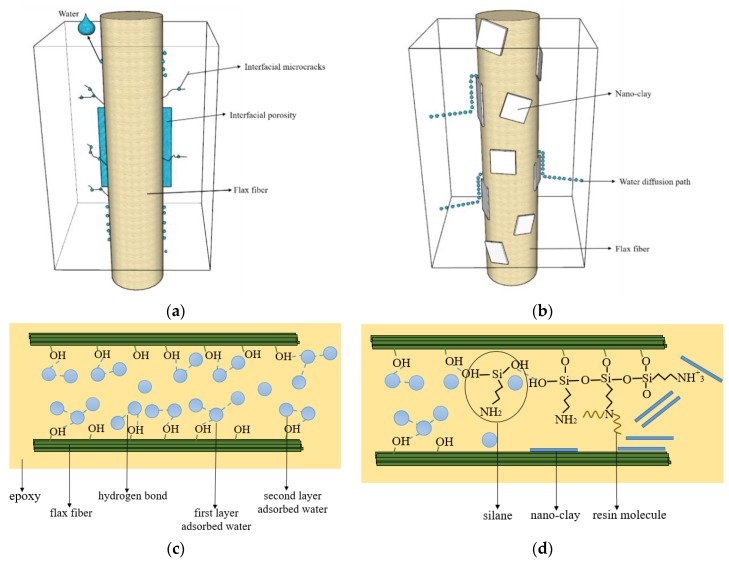
Diagram of water molecule existence and diffusion path in FFRPs (**a**) untreated FFRPs; (**b**) nano-clay grafted FFRPs; model of water molecule interaction on flax fiber-epoxy interface (**c**) untreated flax fibers (**d**) nano-clay grafted flax fibers.

Figure 7a, b show the mechanism of improving the hydrothermal ageing behaviors of nano-clay grafted FFRP. Water molecules enter FFRPs along the fiber direction and are perpendicular to the fiber direction. As shown in Figure 7a, water molecules exist in untreated FFRPs in two forms: (1) free water stored in the cell compartment of fiber, fiber and interfacial space, and micro cracks; (2) bound water adsorbed on the fiber surface and cell wall. As shown in Figure 7b, after the nano-clay grafted, the interfacial adhesion properties of FFRP are improved. Therefore, water molecules in the interfacial and cracks are reduced. The nano-clay is grafted onto the surface of the fiber, making the surface of the fiber hydrophobic, increasing the diffusion path of water molecules. In Figure 7c, water molecules enter the interface between the fibers and epoxy, and form the first layer of water molecules by hydrogen bonding on the surface of unmodified flax fibers. Then the water molecules enter the first layer of water molecules in the form of the second layer of water molecules. As shown in Figure 7d, chemical grafting occupies the hydroxyl groups on the surface of the fiber, reducing the adsorption of water molecules on the cell wall of the flax fiber. However, with the increase of aging time and temperature, water molecules will gradually destroy the chemical bonds between the silane and flax fibers and adsorb on the surface of the fibers.

### 3.3. FBG Monitoring

**Figure 8:**
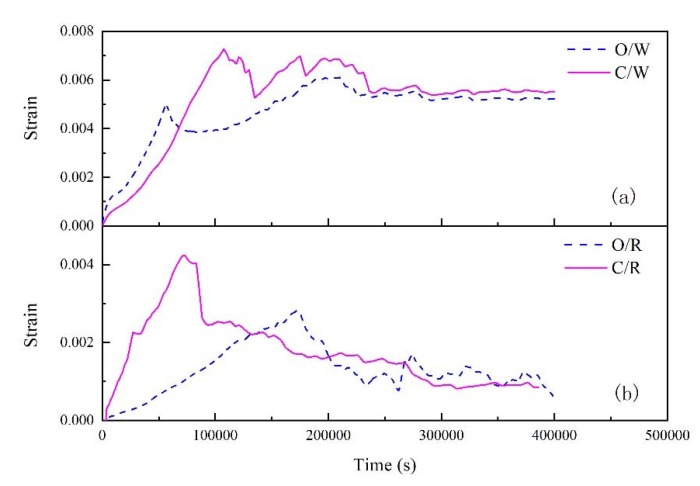
Effect of water absorption on the internal strain of untreated FFRP and nano-clay grafted FFRPs at 70 °C under 80% RH (**a**) the weft strain (**b**) the radial strain.

Figure 8 shows the radial and latitudinal strain values of FFRP calculated by the above formulas, which vary with the time it is placed in the 70 °C under 80% RH environment. Because FBG is sensitive to strain and temperature changes, there are fluctuations in the test results. In order to facilitate the analysis of the results, FBG is used to process the collected data, and the results are simplified to 100 data points without changing the trend. In Figure 8, O/W and C/W represent the weft strain (with less fibers) change process of nano-clay grafted and untreated FFRP; O/R and C/R represents radial strain test results of FBG in nano-clay grafted FFRP and untreated FFRP. The maximum strain in the radial and weft directions of the nano-clay grafted FFRPs are smaller than that of the untreated FFRPs.

As the FFRP is placed in a hygrothermal environment for extended periods of time, water molecules gradually enter the fibers, causing them to expand. With the increase of water absorption, the water molecules diffuse into the specimen through the resin, fibers and the interfaces between them. The water ingress plasticizes the resin matrix, and even the fibers, leading to relaxation of the internal strain [31]. Consequently, the internal tension strain gradually reduces. When FFRP reaches saturated water absorption, the FFRP internal strain tends to balance after a certain fluctuation. As shown in Figure 8, the nano-clay-modified FFRP causes a decrease in the swelling amount of the fiber due to a decrease in saturated water absorption. Nano-clay grafted FFRPs show better dimensional stability.

According to the definition of the linear moisture expansion coefficient, when the laminate absorbs moisture, it produces line strain in the main direction of the material, as per the following formula [32,33]:

(7) $$\beta_{x}=\frac{\varepsilon_{x}}{C}$$

(8) $$\beta_{y}=\frac{\varepsilon_{y}}{C}$$

where $\beta_{x}$ is linear moisture expansion coefficient in the x direction; $\varepsilon_{x}$ is strain in the x direction; $\beta_{y}$ is linear moisture expansion coefficient in the y direction; $\varepsilon_{y}$ is strain in the y direction; *C* is water absorption concentration.

As shown in Table 4, the radial and latitudinal linear moisture expansion coefficient of untreated FFRPs and nano-clay grafted FFRPs are calculated by the maximum strain and saturated water absorption, respectively. The linear moisture expansion coefficient of nano-clay-grafted FFRP in two main directions is smaller than that of untreated FFRP. This is because the presence of the silane coupling agent film and the nano-clay constrains expansion of the fiber.

### 3.4. Tensile Properties

**Figure 9:**
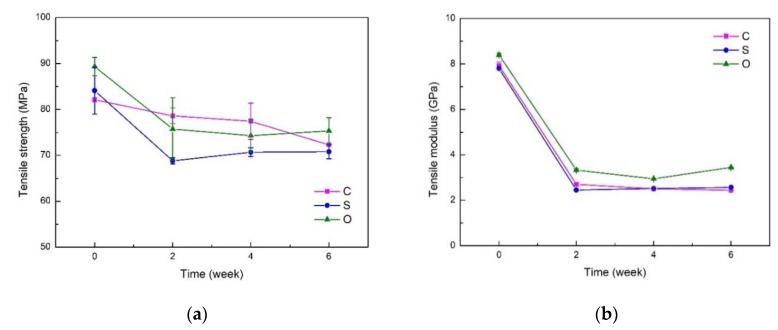
Effect of water absorption on the tensile properties (**a**) tensile strength (**b**) tensile modulus for FFRPs at 70 °C under 80% RH (C: untreated FFRPs; S: silane treated FFRPs; O: nano-clay grafted FFRPs).

Figure 9a,b show the degradation of tensile properties of FFRPs. The degradation of tensile strength is smaller and the degradation of the tensile modulus is larger. The tensile strength of FFRP is affected by the flax fiber strength and the interfacial bond strength between the fiber and resin. The mechanical properties of flax fibers are influenced by the composition, structure and number of defects in a fiber. Under stress, tensile failure occurs by intercellular and/or intracellular modes [34]. Cellular stress is mainly determined by cellulose content and the angle between cellulose microfibers and the axis. When water molecules enter the fibers, moisture in fiber influences the degree of crystalinity and the crystalline orientation of fibers whereby it results in higher amounts and better orientation of crystalline cellulose in fibers. The absorption of water in the pores and amorphous regions of the fibers serves to reduce interfibrillar cohesion and to relieve internal fiber stresses [35]. Cellulose microfibers are embedded in hemicellulose, wax, etc. Hydrogen bonds play a key role in their combination. Water ingress deteriorates the hydrogen bonds, leading to higher elongation and strength, but lower modulus. Besides, increase in tensile strength of flax fiber is due to the availability of free water molecules, providing a plasticizing effect, which is advantageous to the strength of cellulose fibers [15]. However, the plasticization effect of water weakens the fiber/matrix bonding, resulting in interfacial failure [36]. Therefore, when water molecule acts on the composite, the tensile strength of the composite decreases slightly.

As shown in Figure 9a, after a six-week immersion in 70 °C under 80% RH environment, the tensile strength of untreated FFRPs (C) reduced by 13.5%, and that of silane treated FFRPs (S) and nano-clay grafted FFRPs (O) decreased by 15.8% and 15.6% respectively. The tensile strength retention rates of C, S and O were 88.0%, 84.1%, and 84.3%. As mentioned above, the tensile properties of FFRP depend on the tensile strength of the fibers and the strength of fibers depend on the content and angle of cellulose. Thus, surface modification has little effect on the degradation of tensile strength of FFRP in a short time. On the other hand, the interface is the medium of stress transfer between fibers, and also affects the tensile strength of FFRP to some extent. Cellulosic fibers can absorb water from the environment and can swell. This causes shear stress at the interface, which favors ultimate debonding of the fibers, which in turn causes a reduction in tensile strength [36]. The silane coupling agent forms a thin layer of macromolecule at the interface between the fiber and the resin, but the coupling agent is highly sensitive to water molecules [22]. When water molecules enter the early stage of the composite, under the action of high temperature, the Si–O–C bond between fiber and silane is not stable towards hydrolysis [37]. As a result, some of the coupling agent molecules that do not form macromolecules are easily hydrolyzed [38]. After partial hydrolysis of silane coupling agent molecules, the interfacial properties of modified flax fiber composites decreased before those of unmodified composites. Therefore, in the early stage of aging, the tensile strength of the untreated FFRPs decreased less than that of the silane treated FFRPs. For the same reason, the nanoclay grafted FFRP has also been treated with silane coupling agent, so the short-term degradation of tensile properties shows the same regularity as the silane treated FFRP. However, many papers show that the tensile strength of NFRP modified by the silane coupling agent is less than that of the unmodified ones after prolonged soaking time in a hygrothermal environment [22,39,40]. It can be predicted that the tensile properties of the nano-claymodified flax fibers are less degraded under the long-term action of water molecules.

Figure 9b shows the degradation of tensile modulus at 70 °C under an 80% RH environment. The tensile modulus degrades more than the strength. The tensile modulus of untreated FFRPs (C) reduced by 69.2%, and that of silane treated FFRPs (S) and nano-clay grafted FFRPs (O) decreased by 67% and 59.4% respectively. The tensile modulus retention rates of C, S and O were 30.7%, 32.9%, and 41.1%. After the water molecules enter flax fibers, because water molecules can exist in the amorphous structure [8,9], it makes the amorphous structure soften, resulting in a decrease in the modulus of the fiber. Another reason for the decrease in the modulus of the wet sample can be explained by the weakening of the cellulose structure of the natural fiber by the water molecules in the cellulose network structure, where water acts as a plasticizer and allows the cellulose molecules to move freely. Therefore, the quality of the cellulose is softened and the dimensions of the fiber can be easily changed by application of force [41]. The decrease of the absorption of water molecules reduces the plasticizing effect of the composite, so the decrease of the modulus of FFRP grafted by nano-clay is reduced.

**Figure 10:**
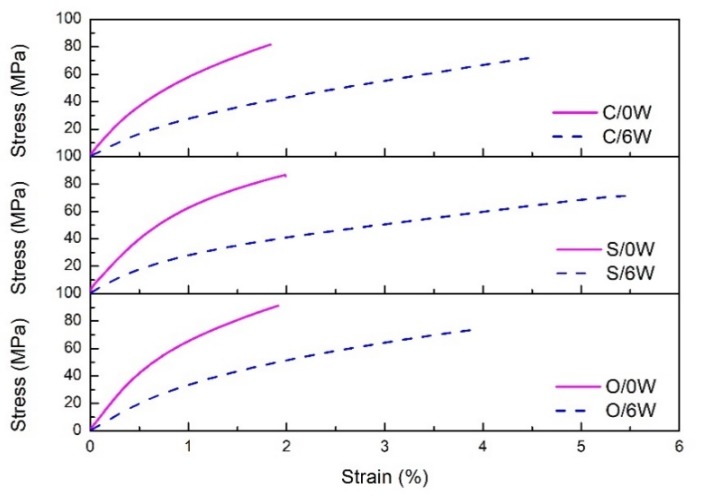
Effect of the water absorption on the stress-strain curves of different FFRPs at 70 °C under 80% RH.

The stress-strain curve of the FFRP tensile test is shown in Figure 10. In this picture, C, S and O represent various FFRP samples. 0W and 6W represent FFRPs before aging and placed at 70 °C under 80% RH environment for 6W, respectively. Curves extended into the nonlinear region in all cases. The stress-strain curve can be divided into three parts: (1) the first linear part, which is the deformation of each cell wall; (2) the second non-linear part, which is the elastic-plastic deformation of the fibers, is the rearrangement of the amorphous part (mainly made of pectin and hemicellulose) in the thickest cell wall (S2); and (3) the final approximately linear part, which is elastic response of cellulose microfibers to applied tensile strain [1]. The elastic linear area, where the damage is irreversible, reduces as a function of the water ageing [42]. The ultimate strain of untreated and nano-clay grafted FFRPs are increased after being placed in hygrothermal environment for 6 weeks. Water molecules can combine with hydroxyl bonds to act as plasticizers, which makes the material more ductile [15]. In addition, the significant increase in failure strain is due to the decomposition of the cellulose structure after the aging process, resulting in increased ductility of the flax fibers [43]. On the other hand, after the water molecules enter the FFRP, they occupy the pores and defects inside, which increases the ultimate strain of FFRP. At the same time, this increase is attributed to the lubrication of the water molecules, which may slide against each other during loading, resulting in more deformation and elongation [44]. As shown in Figure 10, the ultimate tensile strain of the nano-clay grafted FFRP after being placed at 70 °C under 80% humidity environment for six weeks was smaller than that of untreated FFRP. The reason is obvious. The saturated water absorption of the nano-clay grafted FFRP is lower than untreated FFRP, and the plasticization of FFRP by water molecules is reduced, so the increase in ultimate strain is reduced.

**Figure 11:**
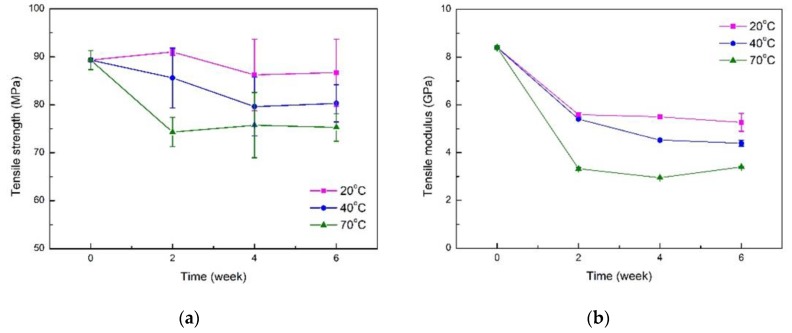
Effect of the water absorption on the tensile properties: (**a**) tensile strength (**b**) tensile modulus for nano-clay grafted FFRPs at different temperature under 80% RH.

Figure 11a,b show the degradation of tensile strength and modulus of nano-clay treated FFRPs in 20, 40 and 70 °C under 80% RH. Similar to the former, FFRPs also show the same degradation law at different temperatures, that is, tensile strength degradation is less, while tensile modulus degradation is greater. With the increase of temperature, the degradation of tensile properties of FFRPs increase, which is due to the increase of temperature, accelerating the movement of water molecules, increasing the diffusion rate of water, accelerating the aging of FFRP. Figure 11a shows the change in tensile strength of nano-clay-grafted FFRP over a six-week period in three different temperatures under 80% RH environments. After six-week immersion in 20, 40 and 70 °C under 80% RH environment, the tensile strength of nano-clay grafted FFRPs (O) decreased by 3.0%, 10.0%, 15.7%. When the nano-clay grafted FFRP is placed in an environment of 20 °C under 80% RH, the tensile strength increases during the first two weeks. Because of the slower diffusion rate of water molecules in a lower temperature, there are a few of water molecules inside the FFRP, and this part of the water molecules enhances the flax fiber without breaking the interface bonding between flax fiber and epoxy. Therefore, the FFRP tensile strength increased during the first two weeks. Subsequently, due to the prolongation of time, the water molecules gradually entered the FFRP, causing the debonding of the interface and damage inside the fiber. Therefore, after four weeks aging, the strength of the FFRP decreased. In addition, due to the difference in the diffusion rate of water molecules and the deterioration of the FFRP at high temperatures, the tensile strength of FFRP does not degenerate after four weeks at 20 and 40 °C under 80% RH, and the FFRP at 70 °C under 80% RH no longer degenerates in two weeks. It is worth noting that although the saturated water absorption of FFRP is approximately the same under different temperature environments, the effects on the tensile properties of FFRP are different. The higher the temperature, the more severe the aging of the composite material. This is because the high temperature accelerates the movement of the water molecules and also increases the deterioration of the composite material. Figure 11b shows the same results, which are the higher the temperature, the greater the modulus drop. After six-week immersion in 20, 40 and 70 °C under an 80% RH environment, the tensile modulus of nano-clay grafted FFRPs decreased by 36.9%, 47.6%, 59.5%.

**Figure 12:**
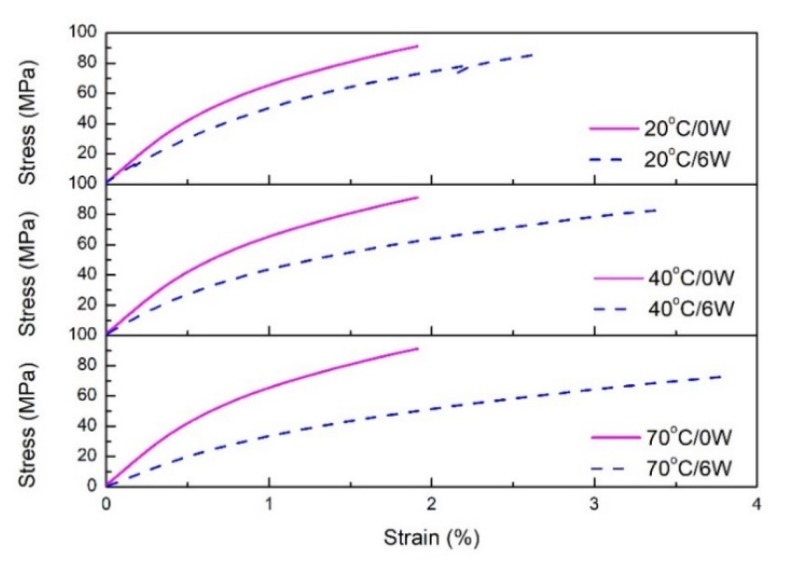
Effect of water absorption on the stress-strain curves of nano-clay grafted FFRPs at different temperatures under 80% RH.

The stress-strain curve of the nano-clay grafted FFRP tensile test after immersion in different environments is shown in Figure 12. Here, 20 °C/0W, 40 °C/0W and 70 °C/0W represent the stress-strain curve of nano-clay grafted FFRP before immersion; 20 °C/6W, 40 °C/6W and 70 °C/6W represent the stress-strain curves of nano-clay grafted FFRP subjected to exposure at 20, 40 and 70 °C under 80% RH for 6 weeks. The result also shows the same result. When FFRP is placed in the hygrothermal environment with a higher temperature, the greater the ultimate strain of FFRP.

Elongation at break of FFRPs show the same results. Table 5 shows the elongation at break of FFRPs. After immersion in 70 °C under an 80% RH environment for six weeks, the elongation at break of FFRPs increases. Among them, the nano-clay grafted FFRP has the smallest change in elongation at break. On the other hand, as the temperature increases, the greater the increase in elongation at break.

### 3.5. SEM Observation

**Figure 13:**
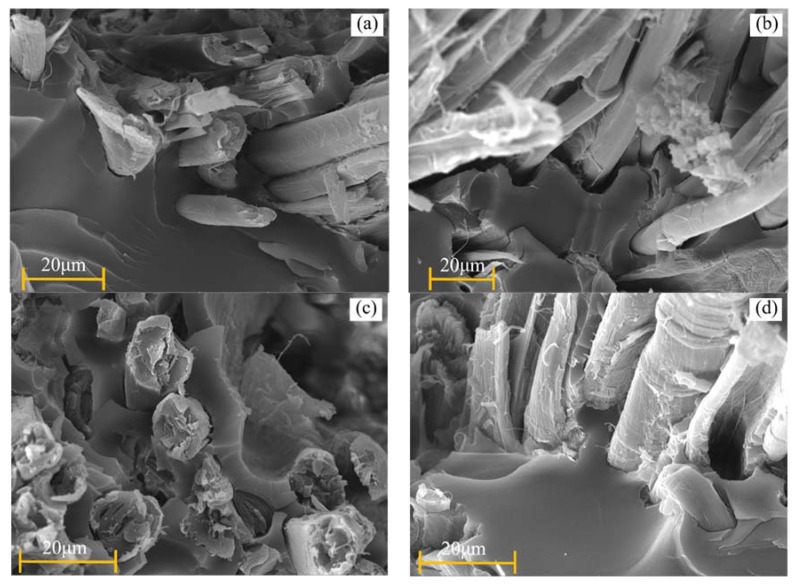
SEM photos of nano-clay grafted FFRPs tensile fracture: (**a**) untreated FFRP before aging; (**b**) untreated FFRP immersion in 70 °C under 80% RH for six weeks; (**c**) nano-clay grafted FFRP before aging (**d**) nano-clay grafted FFRP immersion in 70 °C under 80% RH for six weeks.

Figure 13a–d show the SEM test results of tensile fracture of FFRPs after aging. Figure 13a showed tensile fracture of untreated FFRP before aging in which the fibers were pulled out of the resin but the gap between the fibers and the resin was small. The tensile fracture picture (Figure 13b) of untreated FFRP after six weeks of aging showed that the direct gap between fiber and resin was larger, which indicated that the interface between unmodified fiber and resin was destroyed by water molecules. Figure 13c showed tensile fracture of nano-clay grafted FFRPs before aging. The interfacial adhesion between modified fibers and resins increased. Thus, the interfacial debonding of fiber and resin is less, and most of them are broken by fiber fracture. While after six weeks immersion at 70 °C under 80% RH, nano-clay grafted fibers (Figure 13d) are pulled out of the resin, but there is still a small amount of resin attached to the surface of the fiber, and the gap between the fiber and the resin is small. For the nano-clay grafted FFRPs, the entry of water molecule has a certain effect on the bonding between fiber and resin, but the damage is weaker than that of the untreated ones. Because plant fibers have a multi-stack structure, overall swelling results from the local swelling of each component and each cell-wall layer. As each component of S2 layer has a different swelling behavior, differential swelling stresses may induce structural damage of fibers and thus degrade the mechanical properties.

## 4. Conclusions

In this article, the effects of grafting of nano-clay on the hydrothermal resistance of the flax fiber reinforced epoxy composite plate were investigated. The moisture uptake, dimension change and tensile properties of the composite plates were tested. The following conclusions can be drawn based on the testing results and analysis:

(1) The introduction of nano-clay onto the flax fiber reduced the saturated moisture uptake and the coefficient of diffusion by 38.4% and 13.2% of FFRP compared to the control samples. The introduction of lamellar nano-clay is expected to reduce the hydrophilicity of the fiber surface and increases the diffusion path of water molecules.
(2) Nano-clay grafted FFRPs show better dimensional stability than the untreated ones. The linear moisture expansion coefficient of nano-clay grafted FFRP in radial and weft directions is smaller than that of untreated FFRP.
(3) After exposure for six weeks, the retention rate of the tensile modulus of the nano-clay grafted flax fiber-based FFRP was increased by 33.8% compared with that of the control ones, while the retention rate of tensile strength has a little decrease by nano-clay grafting. On immersion in a hydrothermal environment, the degradation of tensile strength of FFRP is not obvious while the ultimate strain of FFRPs is increased.

## Figures and Tables

**Table 1:** Preparation of a hydrothermal environment with a saturated salt solution.

Temperatures	20 °C	40 °C	70 °C
Saturated salt solvent for 80% RH	Potassium bromide (KBr)	Potassium bromide (KBr)	Potassium chloride (KCl)

**Table 2:** Maximum water uptake and diffusion coefficient (D) of FFRPs at 70 °C under 80% RH.

Samples	M_∞_ (%)	$D\times 10^{-6}\;({\mathrm{mm}}^{2}/s)$
Untreated FFRPs	5.41	3.03
Silane treated FFRPs	4.53	6.04
Nano-clay grafted FFRPs	3.91	2.62

**Table 3:** Maximum water uptake and diffusion coefficient (D) of nano-clay grafted FFRPs at 20, 40 and 70 °C under 80% RH.

Hydrothermal Environments	M_∞_ (%)	$D\times 10^{-6}\;({\mathrm{mm}}^{2}/s)$
20 °C, 80% RH	3.20	0.22
40 °C, 80% RH	4.11	0.43
70 °C, 80% RH	3.91	2.62

**Table 4:** Linear moisture expansion coefficient of FFRPs.

	Maximum Strain (Radial)	Radial Expansion Coefficient	Maximum Strain (Weft)	Weft Expansion Coefficient
Untreated FFRPs	0.0042	0.0777	0.007	0.1296
Nano-clay grafted FFRPs	0.0028	0.0717	0.005	0.1282

**Table 5:** Tensile properties of FFRPs.

Hydrothermal Environments	Properties	Untreated FFRPs		Silane Treated FFRPs		Nano-Clay Grafted FFRPs	
		0W	6W	0W	6W	0W	6W
20 °C, 80% RH	Elongation at break	_	_	_	_	0.88%	1.38%
	Strength (MPa)	82.1	_	84.1	_	89.3	86.7
	Modulus (GPa)	7.9	_	7.8	_	8.4	5.3
40 °C, 80% RH	Elongation at break	_	_	_	_	0.88%	1.85%
	Strength (MPa)	82.1	_	84.1	_	89.3	80.3
	Modulus (GPa)	7.9	_	7.8	_	8.4	4.4
70 °C, 80% RH	Elongation at break	0.84%	2.36%	0.86%	3.51%	0.88%	1.96%
	Strength (MPa)	82.1	72.3	84.1	70.7	89.3	75.3
	Modulus (GPa)	7.9	2.4	7.8	2.6	8.4	3.4
